# Healthcare Waste Generation Rates in the Three Categories of Public Healthcare Facilities in Malawi

**DOI:** 10.1002/puh2.70411

**Published:** 2026-10-14

**Authors:** Grace O. Ogbonna, Russel C. G. Chidya, Brighton A. Chunga, Rosemary C. Ogbonna, Fanuel Kapute, Thokozani Mzumara

**Affiliations:** ^1^ Mzuzu University Mzuzu Malawi; ^2^ Abia State Specialist Hospital Umuahia Nigeria; ^3^ Optometry Unit Department of Curative and Rehabilitative Services Mzuzu Urban Community Hospital Mzuzu Malawi

**Keywords:** healthcare waste budgeting, healthcare waste management, Malawi, planning, waste generation rates

## Abstract

**Background:**

The assessment of the waste generation rate is an initial and critical step in addressing poor healthcare waste management practices.

**Aim:**

This study aimed to determine the healthcare waste generation rates in primary, secondary, and tertiary healthcare facilities in Malawi.

**Methods:**

This cross‐sectional quantitative study was conducted in two tertiary (Kamuzu and Mzuzu Central Hospital [MCH]), secondary (Nkhata Bay and Rumphi), and primary (Bolero and Chintheche Rural) healthcare facilities. The broad categorization of healthcare waste into hazardous (sharps, infectious, general, and expired drugs) and nonhazardous (general) wastes was adopted. The waste was measured using a digital scale, and the waste generation rate was subsequently determined. The waste generation measurements were conducted for 28 days. The data were analyzed using R version 4.2.0. MANOVA was used to compare the waste generation means among the healthcare categories at a 5% level of significance.

**Results:**

The results showed that the overall waste generation rates based on bed occupancy and the number of patients seen were varying. MCH (1.07 kg/bed/day) had the highest, whereas Bolero Rural Hospital (0.06 kg/bed/day) had the lowest waste generation rates. The tertiary hospitals generated more waste (35,659.68 kg), including hazardous (22,223.33 kg) and nonhazardous (13,447.09 kg) waste, than the other healthcare categories. There was a significant difference (*p* < 0.001) in the waste generation rate based on the category of healthcare facility.

**Conclusion:**

The waste generation rate was within the range reported for most low‐income countries. However, hazardous wastes (62.26%) were generated, above the World Health Organization (WHO) recommended amount (<25%), thereby posing a risk for increased waste management costs and occupational hazard occurrence. This study highlights the need for concerted efforts to reduce the amount of hazardous waste generated.

## Graphical abstract

Two tertiary (Kamuzu and Mzuzu Central), secondary (Nkhata Bay and Rumphi), and primary (Bolero and Chintheche Rural) healthcare institutions participated in a study on healthcare waste. Waste was categorized as nonhazardous or hazardous. Significant differences in waste generation rates were observed on the basis of facility type (*p* < 0.001).

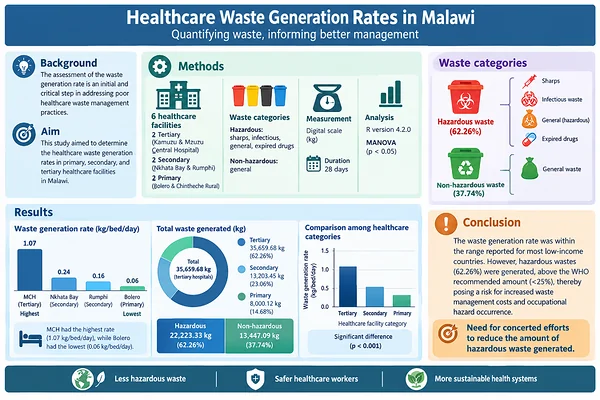

## 1 Background

Healthcare is a recurring need for current and future generations [1, 2, 3]. In providing health services, solid waste, also known as healthcare waste, is generated [4, 5]. Hospital waste is a significant source of health hazards for healthcare personnel, the general public, and the environment at large [6]. Approximately 75%–90% of healthcare waste has similar characteristics to domestic waste and is considered nonhazardous, whereas 10%–25% has hazardous properties and can cause harm to people and the environment [7]. Healthcare waste is responsible for several detrimental effects, such as air pollution, soil, and groundwater pollution, besides causing disruptions to the ecosystem and contributing to climate change [8].

Globally, an increasing amount of healthcare waste is generated daily. Hospitals across the globe generate millions of tons of waste on a daily basis [8]. Approximately 7–10 billion tons of healthcare waste are generated annually [9]. Countries, such as the United States and India, generate approximately 3.5 and 0.33 million tons of healthcare waste annually, respectively [6, 10, 11]. In South Africa, 42,000 t of healthcare waste were generated in 2010 [12]. Tanzania, which shares similar sociodemographic features with Malawi, is reported to have a lower waste generation rate [13]. In Malawi, as in most countries in the Global South, there is a dearth of information regarding waste generation [14]. However, in light of the growing population, the daily generation rate of healthcare waste across the country is expected to increase for both nonhazardous and hazardous materials, which can be harmful to people and the environment at large.

The healthcare waste generation rate is considered an indicator of a hospital's waste management performance [15] and is measured in units of mass in kg per bed per day (kg/bed/day) [16] or kg/patient/day [15]. Factors, such as increases in population, life expectancy, and hospital usage secondary to the occurrence of communicable and noncommunicable diseases, increase healthcare waste generation [17]. Other factors, such as the type of hospital, department, services provided, total number of beds, bed occupancy, number of infectious disease beds, and number of outpatients per day, also affect waste generation [18, 19]. Furthermore, factors, such as per capita income, population growth, urbanization, and industrialization, also contribute significantly to the quantity of healthcare waste generated in a country [15]. This therefore leads to variance in the inter‐ and intrahospital waste generation rates. For example, Komilis [20] observed variations in the waste generation rate between different wards (birth, cancer treatment, general, military, pediatric, and psychiatric) and types of clinic ownership (e.g., private and public hospitals) in their study of 132 healthcare facilities.

Several studies have reported varying waste generation rates in different countries. Hasan and Rahman [21] reported overall waste generation rates and hazardous waste rates of 0.90 kg and 0.18 kg/bed/day, respectively, in Khulna city, Bangladesh. A study in Ethiopia reported a waste generation rate of 0.02–0.03 kg/patient/day in health centers and 1.23 kg/bed/day in a referral hospital [22]. In another study conducted at the Menelik II referral hospital in Ethiopia, a mean waste generation rate of 1.94 ± 0.0335 kg/bed/day was reported [23]. The study also revealed that 50.1% of the generated waste was hazardous waste compared with general waste.

With its growing population and rapid increase in the number of people with communicable and noncommunicable diseases [24], Malawi's public healthcare system is predominantly categorized into primary, secondary, and tertiary healthcare systems. Each level offers a different level of healthcare service delivery to clients. This categorization focuses more on functional levels of care (primary, secondary, and tertiary) rather than a strict geographic district–regional–central structure, with patients often going straight from district to central hospitals because there is not a strong regional layer. In Malawi, this is largely due to limited human and material resources; by implication, central hospitals serve as both regional and national referral centers. This system is also heavily shaped by the donor system and the need to strengthen the primary health centers while reducing the complexity of hospital hierarchies.

Most countries have published literature on waste generation [25, 26, 27], but there are currently no studies focusing on the country of Malawi. Therefore, this study aimed to determine the rates of healthcare waste generation at primary, secondary, and tertiary healthcare facility categories in Malawi. Assessing waste generation rate is an initial and critical step in addressing poor health planning and budgeting for waste management activities, such as collection, transportation, treatment, and disposal. Although there may be similarities between countries’ waste generation rates, relying on data from other countries for planning in Malawi is not ideal because it is not clear what relationships exist between national and subnational levels in the country in relation to planning, policymaking, and target setting [28]. Moreover, the World Health Organization (WHO) advocates for each country to have its own data, given the peculiarities specific to each nation, especially in light of Malawi's vision of 2063 that is specific to it. Information on the waste generation rate is vital for planning and designing integrated healthcare waste management systems. It is also vital for appropriate resource allocation and policy implementation.

## 2 Materials and Methods

### 2.1 Study Design and Area

The study was designed as a multicenter descriptive cross‐sectional study using a quantitative research approach. The study was conducted in two tertiary hospitals, namely Kamuzu Central Hospital (KCH) and Mzuzu Central Hospital (MCH), and two secondary hospitals, including Nkhata Bay District Hospital (NDH) and Rumphi District Hospital (RDH). Primary facilities were Bolero Rural Hospital (BRH) and Chintheche Rural Hospital (CRH) healthcare facilities. The secondary and primary healthcare facilities were selected from the northern region. The two tertiary hospitals are the only referral hospitals in the northern and central regions of Malawi. The KCH is located in Lilongwe district and serves the central region, including the capital city Lilongwe, whereas MCH is located in Mzimba district and serves as the referral hospital for the northern region, including Mzuzu City. According to the 2018 Population Census report, Malawi has a total population of 17.6 million people (17,563,749) (NSO, 2018), with an average growth rate of 2.9%. The populations of the northern and central regions are 2,289,780 and 7,523,340, respectively [27]. These regions contribute 13% and 42.8%, respectively, to the population of the country.

### 2.2 Data Collection Methods and Tools

The broad categorization of healthcare waste into hazardous waste types (sharps, infectious, and expired drugs) and nonhazardous waste types (general waste) was adopted in this study [29]. An observational method was used to identify waste on the basis of its category and the sources of waste on the basis of hospitals and departments. Although the Malawi Infection Prevention and Control guidelines adopt the standard WHO healthcare waste color‐coding system [30], what we observed in the study facilities was slightly different. Wastes were segregated as follows: sharps were disposed of in yellow safety boxes; infectious and highly infectious waste was collected in yellow or red bins; and general waste was disposed of in green or black bins, and expired drugs were stored in cartons. For this study, the observed practice was standardized as follows: yellow safety boxes marked as sharps were used for the collection of sharps waste, yellow bins for infectious and highly infectious wastes (which were mixed in the hospitals), black bins for general waste, and brown bins for expired drugs.

The wastes were bagged in color‐coded leakproof bin bags and measured using a digital scale. The measured wastes were thereafter recorded in a data entry sheet in kilograms (kg) according to their category against the department where they were generated. The measured wastes were also recorded according to their category against the department where they were generated. To ensure adequate quantification of waste, every waste generated was measured at its source department or unit from which it was generated and recorded. Waste measurements were performed at the same time every day (at the time when waste was removed from their sources, e.g., in the morning and in the evening). Waste generation for each unit per day was measured for a period of 28 days and calculated using the method described by Malekahmadi and Yunesian [31].

Details on the number of beds available in the hospital by ward and department were obtained from the records department via the administrative office. The occupancy rate was determined by knowing the number of beds occupied each day (beds not occupied were not counted, and patients who used methods other than beds were counted as using a bed). Bed occupancy was regarded as a full day cycle, for example, 24 h of bed usage. Due to the shortages of beds and the overflow noted in the facilities, bed occupancy was calculated on a 28‐day basis to get an average bed occupancy rate. The number of outpatients visiting the hospital was also determined daily through records from the outpatient department as submitted to the records department. At the end of the weighing period, the average weight of the waste generated based on waste type was taken as the daily average. The tools for data collection included a digital scale (TCS 500 kg, price scale), a camera, a sharp box and bin liners (waste disposal bags), and a data entry sheet. The data were collected for 28 days (August 23 to September 20, 2021).

### 2.3 Data Management and Statistical Analysis

The data were entered into an Excel spreadsheet and exported to R version 4.2.0 for analysis. Daily bed occupancy and patient inflow were used to determine waste generation rates within healthcare facilities [15]. The following formulas were applied as follows:

- Total waste generation rate = total waste generated
- Total daily waste generation rate (waste generation rate per day) = sum of waste generated (total kg of waste generated)/number of days of measurements
- Waste generation rate per patient per day = total waste generated/number of days × average of the total number of patients seen
- Waste generation rate per bed per day = total waste generated/number of days × average of the total number of beds occupied.

MANOVA was used to compare the waste generation means among different hospitals, waste types, and hospital categories at a 5% level of significance. Significantly different waste generation means were separated using Tukey's multiple comparisons test. The results are summarized and presented in tables.

### 2.4 Data Quality Assurance

Training was given to the data collectors and supervisors before the data were collected. Weighing scales were digitalized and calibrated before measurements were taken either in the morning or evening. In addition, the weights of the waste receptacles (e.g., bin bags) were tared into the scales before the measurements were taken. Furthermore, daily on‐site supervision of the measurement process was provided by trained supervisors.

## 3 Waste Generation Rate in Healthcare Facilities

With a total of 2082 beds, an average occupancy rate of 100% across 28 days, and an average daily patient load of 5572 across all the healthcare facilities (Table 1), a total waste generation rate of 40,008.98 kg was registered. The total daily waste generation rate was 1428.89 kg/day. Hazardous waste (62.26%; 24,911 kg) was generated more than nonhazardous waste (37.74%; 15,097.73 kg), with infectious wastes, sharps, and expired drugs contributing 83.97%, 10.18%, and 5.85%, respectively, to the total hazardous waste composition. Furthermore, on the basis of bed occupancy and the number of patients attending clinics, the waste generation rates for the facilities were determined to be 0.69 kg/bed/day and 0.26 kg/patient/day, respectively. Furthermore, on the basis of the bed occupancy rate, MCH had the highest waste generation rate of 1.09 kg/bed/day, whereas the lowest was recorded in BRH (0.07 kg/bed/day). On the other hand, KCH generated the greatest amount of waste (24,209.02 kg), whereas Bolero Rural Hospital generated the least amount of waste (236.108 kg) and the lowest waste generation rate from patient inflow (Table 2). Multiple comparisons of the means (Turkey contrast) showed that the waste generation rates were not significantly different between all the facilities except for KCH, where the rates differed markedly (<0.001).

**TABLE 1:** Inpatient bed capacity and average daily outpatient attendance across facilities.

Facility	Total inpatient beds	Total patients (28 days)	Average daily outpatients
KCH	918	50,162	1792
MCH	374	28,384	1014
NDH	350	27,600	986
RDH	350	28,101	1004
CRH	30	18,180	649
BRH	60	3601	129
**Total**	**2082**	**156,028**	**5572**

Abbreviations: BRH, Bolero Rural Hospital; CRH, Chintheche Rural Hospital; KCH, Kamuzu Central Hospital; MCH, Mzuzu Central Hospital; NDH, Nkhata Bay District Hospital; RDH, Rumphi District Hospital.

**TABLE 2:** Waste generation rate across healthcare facilities.

Facility	Total waste (kg)	Daily waste (kg/day)	Waste rate (kg/bed/day)	Waste rate (kg/patient/day)
KCH	24,209.72	828	0.94	0.48
MCH	11,460.60	401	1.09	0.40
NDH	1755.07	65.97	0.18	0.06
RDH	1964.45	65.30	0.20	0.07
CRH	383.04	12.31	0.46	0.02
BRH	236.10	8.27	0.14	0.07
**Total**	**40,008.98**	**1428.89**	**0.69**	**0.26**

Abbreviations: BRH, Bolero Rural Hospital; CRH, Chintheche Rural Hospital; KCH, Kamuzu Central Hospital; MCH, Mzuzu Central Hospital; NDH, Nkhata Bay District Hospital; RDH, Rumphi District Hospital.

### 3.1 Waste Generation Rate by Category of Healthcare Facility

There was a significant difference (*p* < 0.001) in the waste generation rate based on the healthcare facility category assessed. The tertiary hospital recorded the highest quantity of waste (35,669.62 kg) and waste generation rate (0.45 kg/patient/day). On the other hand, the primary health centers generated the least amount of waste (619.14 kg), with a waste generation rate of 0.03 kg/patient/day. Multiple comparisons of the means (Turkey contrast) revealed that the waste generation rates were statistically similar between the primary and secondary healthcare facilities but differed between the tertiary health facilities (Table 3).

**TABLE 3:** Waste generation rates by hospital category.

Hospital	Total (kg)	Waste generation rate (kg/px/day)	Waste generation rate (kg/bed/day)	*p* value
Primary	619.137	0.03	0.3	<0.001
Secondary	3719.52	0.07	0.19	
Tertiary	35,670.32	0.45	1.02	

*Note:* *p* < 0.05 is statistically significant.

Abbreviation: kg, kilogram.

### 3.2 Waste Generation Rate by Type of Waste in the Different Categories of Healthcare Facilities

On the basis of the type of waste, the healthcare facilities generated more hazardous (24,911 kg) than nonhazardous waste (15,097.73 kg), contributing 62.26% and 37.74%, respectively, to the total waste generated. Furthermore, tertiary hospitals generated the greatest quantities of infectious (19,571.02 kg), noninfectious (13,447.09 kg), and sharp waste (1405.01 kg). On the other hand, the primary health centers generated the least amount of noninfectious (267.67 kg), infectious (170.47 kg), and sharp waste (139.73 kg). Multiple comparisons of the means (Turkey contrast) revealed that the waste generation rates were statistically similar between the primary and secondary healthcare facilities but differed between the tertiary health facilities. This difference was also found to be statistically significant (Table 4). Furthermore, the study revealed a similar percentage contribution of hazardous waste to the total waste volume in tertiary (62.30%) and secondary (62.81%) healthcare facilities, which was different from that in primary healthcare facilities (56.76).

**TABLE 4:** Waste generation by type of waste based on the category of the healthcare facility.

Hospital	Infectious (kg)	Noninfectious (kg)	Sharps (kg)	Expired drugs (kg)	Total (kg)	*p* value
Primary	170.49	267.67	139.73	41.25	619.13	**<0.001**
Secondary	1177	1382.97	991.44	167.85	3719.52	
Tertiary	19,571.02	13,447.09	1405.01	1247.2	35,670.32	

*Note:* *p* < 0.05 is statistically significant.

Abbreviation: kg, kilogram.

### 3.3 Waste Generation Based on the Category of Wastes in Different Healthcare Categories

Further analysis revealed that tertiary healthcare facilities generated more hazardous waste (22,2 kg) compared to the other healthcare categories (*p* −0.001).

## 4 Discussion

Waste generation rates vary from one facility to another, within a country and between countries [32]. These differences highlight the effects of population, size, the availability of funds, seasonal changes, and waste management practices on the waste generation rate [33]. Although many studies have been conducted to review the waste generation rate in different countries, in Malawi, this is the first study to evaluate the healthcare waste generation rate in healthcare facilities.

The current study found that the overall waste generation rate by bed occupancy was determined to be 0.69 kg/bed/day. This waste generation rate is low but within the expected rates for most low‐income countries. Several studies have reported that waste generation rates are lower in middle‐ and low‐income countries than in high‐income countries [34]. Compared to the findings of the current studies, countries, such as Pakistan,  Lebanon, and Iran, reported higher waste generation rates between 0.3–2, 2.5 kg/bed/day, and 4.6kg/bed/day, respectively [33, 34, 35, 36]. However, this value is greater than the 0.179 kg/bed/day observed in a teaching hospital in India [37]. The differences in waste generation rates may be a reflection of the differences in funding as well as in the economic status of the countries [34]. The variations may also be due to the exclusion of primary and secondary healthcare facilities in the other studies. Additionally, given that the current study was conducted at the tail end of the COVID‐19 pandemic, the influence of the measures implemented during the pandemic to minimize spread may have affected the waste generation rate.

Similar to a previous study [38], the current study revealed that the waste generation rate by rate of bed occupancy was greater than the rate of generation by patient attendance. This can be explained by the fact that there are often more patients than there are beds in most hospitals in low‐income countries. This is a reflection of the low funding challenges often plaguing healthcare in Africa.

Variations were observed between the different categories of healthcare facilities. Significant differences were observed in the waste generation rates between tertiary hospitals and the other healthcare categories studied. At the tertiary level, the waste generation rate was higher than what was reported in both the secondary and primary healthcare facilities. The current study revealed the lowest waste generation rate of 0.12 kg/patient/day at the primary healthcare level. The primary healthcare facility is the healthcare facility at the grassroots level and often serves as the point of contact for patients seeking healthcare services. Hence, it provides nonspecialized services to the members of the community. Given the scope of the service that it provides, primary healthcare services are often smaller than secondary and tertiary healthcare services [22]. As such, the smaller waste generation rates reported by the primary healthcare facilities compared to other categories of healthcare facilities were within reason.

The current study revealed a higher waste generation rate (1.02 kg/bed/day) at tertiary healthcare facilities than at the referral hospitals in Ethiopia and Bangladesh (0.866 and 0.90 kg/bed/day [21, 39], respectively). However, this differs markedly from the waste generation rate observed in other tertiary hospitals in countries, such as China (1.22 kg/bed/day) [40], Pakistan (3.7 kg/bed/day) [41], and Nigeria (2.5 kg/bed/day) [33]. Furthermore, the current study revealed that the daily waste generation rate per patient was 0.45 kg/px/day. This value is lower than the 1.70 kg/patient/day reported in Ghana [42].

Studies have shown that there is a positive relationship between HCW generation rates and gross domestic product (GDP), life expectancy, the human development index, and health expenditure by a country [16]. Hospitals within Malawi are funded by donors and government allocations, with less than 20% of the total revenue generated through out‐of‐pocket payment and health management organizations [43]. This implies that revenue for the procurement of necessary medical resources may be dependent on the availability of funds by the government or through donors. The waste generation rate observed in this study may point to the need for greater funding from healthcare facilities in Malawi.

Tertiary hospitals in Malawi are apex hospitals and may enjoy greater resource allocation and donor‐driven resource mobilization than secondary and primary healthcare centers. These differences showcase the variation that exists between hospitals within the same country. Similar variations were reported in studies conducted among tertiary hospitals in China and Egypt. Such variation may be due to the increasing use of disposable items. This may also be an indication of waste management practices in different facilities.

Contrary to the study by Mekonnen et al., where the majority of the waste generated at healthcare facilities was composed of nonhazardous waste [44], the current study revealed the generation of a greater quantity of hazardous waste than nonhazardous (general) waste. The quantity of hazardous waste generated from healthcare facilities was nearly three times the upper limit of hazardous waste that could be generated from healthcare facilities that practice adequate waste segregation, as estimated by the WHO. A contrasting study in Addis Ababa, Ethiopia, revealed that hospitals produce more general waste [45]. The current findings also differ markedly from those reported in a teaching hospital in Ghana, where general waste constituted approximately 56% of the total waste [42].

The tertiary healthcare facilities generated more hazardous waste than did the other healthcare facility categories. However, when further analyzed, the study revealed similar percentage contributions of hazardous waste to the total waste volume in secondary and tertiary healthcare facilities that were different from what was observed in primary healthcare facilities. Notably, primary healthcare facilities have departments and wards that are different from what is obtainable in secondary and tertiary healthcare facilities; hence, it is expected that the types of waste generated from these facilities should also differ. This is in tandem with the findings of the study by [22] in Ethiopia, where a tertiary hospital generated more hazardous waste than did primary healthcare centers. Although the study by [22] did not involve secondary healthcare facilities, it highlights the differences in the quantity of waste generated in healthcare facilities based on their waste types. It is also a reflection of the advanced services that are accessible at tertiary hospitals. It may also be a reflection of their waste management knowledge and waste segregation practices.

Although general waste comprises 75%–90% of the total waste generated in healthcare facilities, when it is stored together with infectious or other hazardous wastes, it is treated in the same manner as hazardous waste [46]. Observations showed that most of the wastes were general wastes (e.g., kitchen wastes, wood, paper, cans, cardboard boxes, cartons, plastics, and packaging materials) mixed in the same waste bins as infectious wastes (blood‐stained cotton swabs, urine bags, bandages, diapers, catheters, tubing, surgical gloves, nose masks, syringes without needles, and human anatomical wastes). Notably, an increase in the volume of hazardous waste increases the cost of waste management. For example, it was reported that a 20% decrease in the hazardous waste generation rate resulted in $11.8 million saved in the United Kingdom [19]. The quantity of hazardous waste reported in this study may be a direct reflection of the poor knowledge and waste management practices as observed by Alam et al. [47]. This may also have a direct impact on the high prevalence of occupational hazards and can contribute to pollution as well as disrupt the ecosystem.

### 4.1 Limitations

The study is not without limitations. The use of MANOVA instead of a generalized linear mixed model could affect repeated measures on the same facility; MANOVA assumes independence, which may be violated in the study. Secondly, the study sites were limited to a small number of facilities, which could not be representative of many facilities in the country, hence affecting generalizability. Furthermore, private hospitals were not considered in the study, also affecting generalizability of the findings. Moreover, the study did not report the full post hoc Tukey output (mean difference, 95% CI, adjusted *p*) for the pairwise comparisons, leading to uninformative “significantly different” without effect size. Finally, the study did not categorize hospitals according to department to provide more insights into waste generation rates according to department.

## 5 Conclusion and Recommendations

The current study found that healthcare waste generation rates were within the range commonly reported for low‐income countries. However, the proportion of hazardous waste exceeded the recommended levels, indicating challenges in waste segregation practices across the healthcare facilities. The mixing of general and infectious waste not only increases the volume that requires specialized treatment, but it also raises the management costs and heightens the risk of occupational exposure among healthcare workers. It also reflects potential gaps in staff training and compliance with established guidelines. These challenges may be further compounded by resource constraints and reliance on donor funding, which can affect the consistency and sustainability of waste management practices. There is a need for *q* longitudinal and multicenter studies to better understand seasonal trends in waste generation and the impact of policy implementation over time in public healthcare facilities across Malawi. Additionally, research exploring the economic burden of poor waste management, as well as staff knowledge and behavioral factors influencing segregation practices, would provide valuable insights for strengthening healthcare waste management systems.

## Author Contributions

Grace O. Ogbonna was responsible for the drafting of the manuscript, conceptualization of the study, and data collection and analysis. R.C.O. was responsible for the data analysis, study conceptualization, manuscript drafting, and editing. R.C.G C., Fanuel Kapute, and Brighton A. Chunga were responsible for the supervision of the study, manuscript drafting, and editing of the study. Thokozani Mzumara was involved in writing and reviewing the manuscript, data analysis, and interpretation of the study.

## Funding

The authors have nothing to report.

## Ethics Statement

Ethical approval to conduct this study was obtained from the institutional review board of Mzuzu University, “Mzuzu University Research Ethics Committee,” MZUNIREC/DOR/21/42. Similarly, permission to conduct the study was obtained through the Research Ethics Committee of the respective hospitals involved in the study prior to the start of the study. The study adhered to the tenets of the Declaration of Helsinki.

## Consent

The authors have nothing to report.

## Conflicts of Interest

The authors declare no conflicts of interest.

## Data Availability

The datasets used and analyzed during the current study are available from the corresponding author upon reasonable request.
